# Supplementary material for: Inward motion of diamond nanoparticles inside an iron crystal
Source: Nat Commun. 2024 May 31;15:4659. doi: 10.1038/s41467-024-48692-5 (PMC11143255; doi:10.1038/s41467-024-48692-5)
Supplement: Supplementary file 1 — Supplementary Information [file 41467_2024_48692_MOESM1_ESM.pdf]

Supplementary Information
for
Inward motion of diamond nanoparticles inside an iron crystal

Yuecun Wang[1*], Xudong Wang[2*], Jun Ding[2*], Beiming Liang[1], Lingling Zuo[1], Shaochuan Zheng[1], Longchao Huang[1], Wei Xu[1], Chuanwei Fan[1], Zhanqiang Duan[3], Chunde Jia[3], Rui Zheng[1], Zhang Liu[1], Wei Zhang[2], Ju Li[4], En Ma[2†], Zhiwei Shan[1†]

[1]Center for Advancing Materials Performance from the Nanoscale (CAMP-Nano) & Hysitron Applied Research Center in China (HARCC), State Key Laboratory for Mechanical Behavior of Materials, Xi'an Jiaotong University, Xi'an 710049, China

[2]Center for Alloy Innovation and Design (CAID), State Key Laboratory for Mechanical Behavior of Materials, Xi'an Jiaotong University, Xi'an 710049, China

[3]Department of Materials Science and Engineering, Shenyang Ligong University, Shenyang, 1100159, China

[4]Department of Nuclear Science and Engineering, and Department of Materials Science and Engineering, Massachusetts Institute of Technology, Cambridge, Massachusetts 02139, USA.

Supplementary Figures

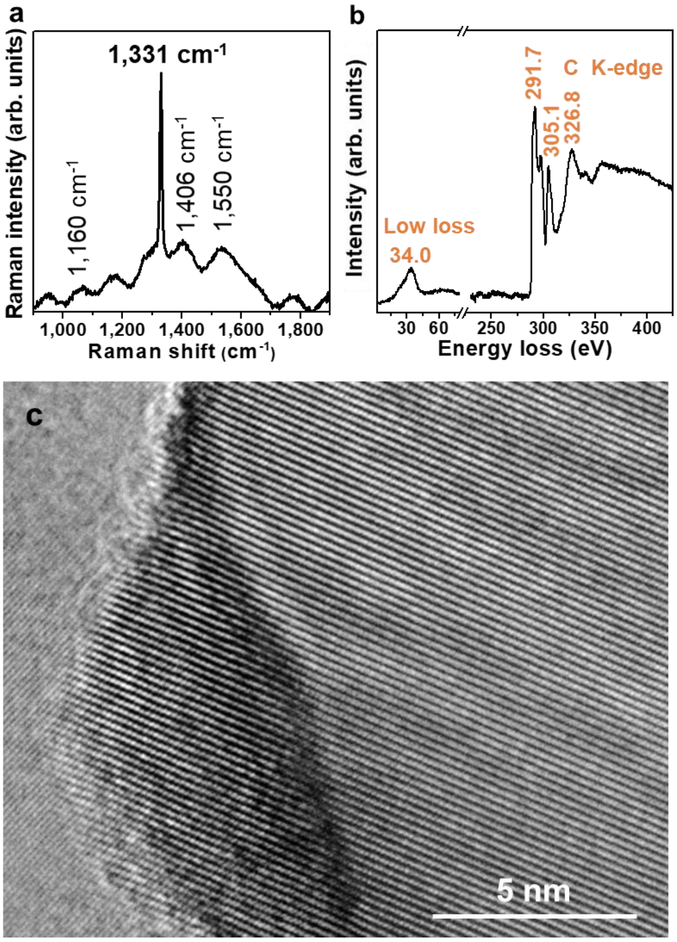

Supplementary Figure 1. Characterizations of DNPs. a A typical Raman spectrum of DNPs on pure iron**. b** Low-loss (plasmon peak at ~34 eV) and core-loss EELS spectra taken from DNPs, verifying that the carbon mainly is *sp*^{3}-hybridized**. c**, A representative high-resolution TEM image of DNPs (with a thin amorphous carbon layer on the surface).

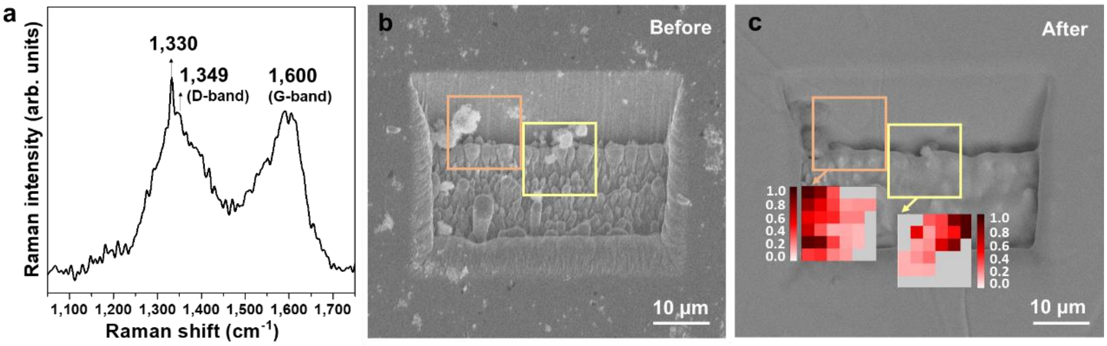

Supplementary Figure 2. Post-mortem Raman spectroscopic characterizations on the sample after in-situ heating treatment inside SEM. a A representative Raman spectrum acquired from a heated iron sample. **b** An iron sample (before it was heated) with a zone delineated by FIB milling, containing blow-spread DNPs. **c** The normalized

intensity maps of the nanodiamond characteristic peaks (~1,330 cm^{-1}) in the Raman spectra acquired from two regions of interest, framed by orange and yellow boxes.

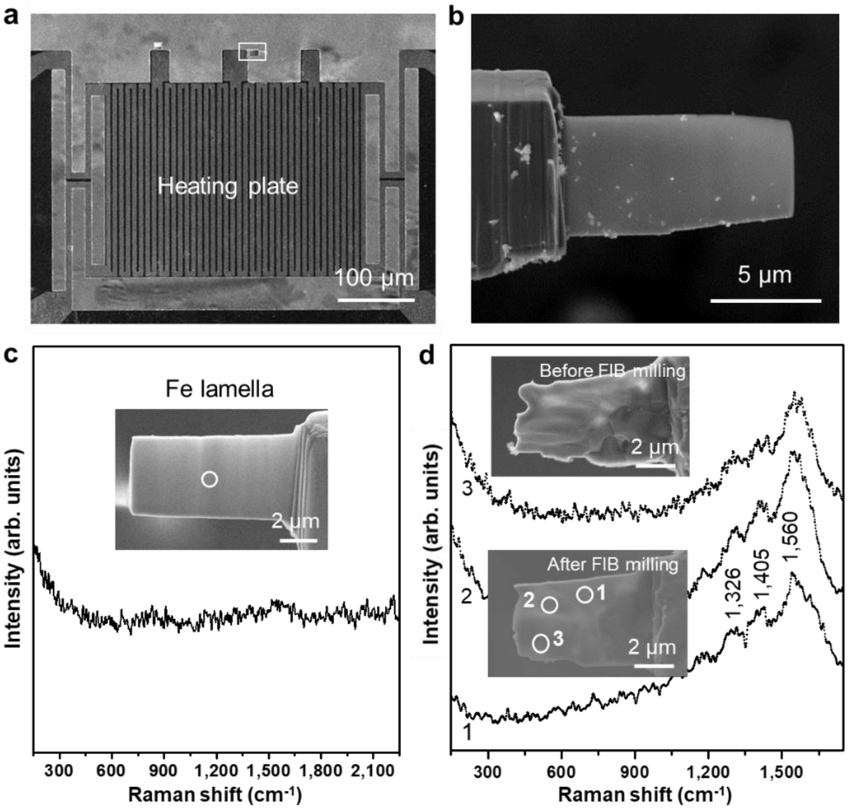

Supplementary Figure 3. Sample and setup for the in-situ TEM heating experiments. a SEM image of the home-made MEMS heating chip with the heating/sensing traces and sample mounting bars in the hotplate of the chip. **b** Enlarged SEM view of the area framed by white rectangle in a, demonstrating a lifted-out pure Fe lamella with scattered DNPs on its surface. **c** A Raman spectrum from the circled area on the as-fabricated Fe lamellae (without DNPs). **d** Raman spectra acquired from three circled areas (numbered as 1, 2, 3) on the heated Fe lamella with DNPs. Post-mortem SEM characterizations after cooling down to RT (insets) indicate that the sample indeed became thicker at the location where the DNPs were swallowed. To remove the possible residual DNPs on the surface and expose the interior DNPs, FIB-milling was performed on the lamella. For the Raman spectra acquired from in-situ TEM samples (c, d), the intensity of the diamond characteristic peak is low, since only a small number of DNPs were blow-spread onto the Fe for the convenience of real-time observations. Besides, a fraction of the DNPs engulfed by Fe released some carbon atoms from their amorphous and graphitized surfaces. At the carburizing temperature the carbon atoms dissolve into Fe to satisfy the relatively large equilibrium solubility, forming no carbide at the Fe-graphite interface. Upon cooling down to RT later, the Fe-C solid solution becomes supersaturated, such that in some sample regions small carbides or graphite precipitates have been observed. This explains why the Raman peak intensity of graphite at 1,560 cm^{-1} is high. The spectrums also shows the presence of nanoscale carbide indicated by the weak peaks between 200 and 600 cm^{-1} [1,2].

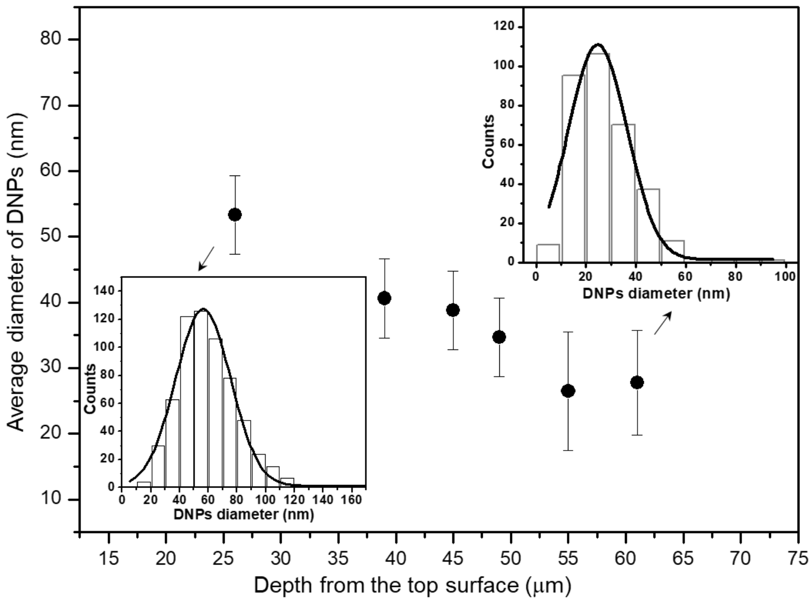

Supplementary Figure 4. The average size of DNPs inside iron decreases with the increased depth from the top surface of sample. The left bottom and right upper insets show the Gaussian distribution of DNPs size at the depth of 26 μm and 61 μm, respectively. DNP sizes (i.e., diameters of the circular cross-sectional area) were measured manually from high-resolution SEM images of the deeply etched samples (treated in furnace for 24 hours). The error bars indicate statistical errors in terms of the standard deviations.

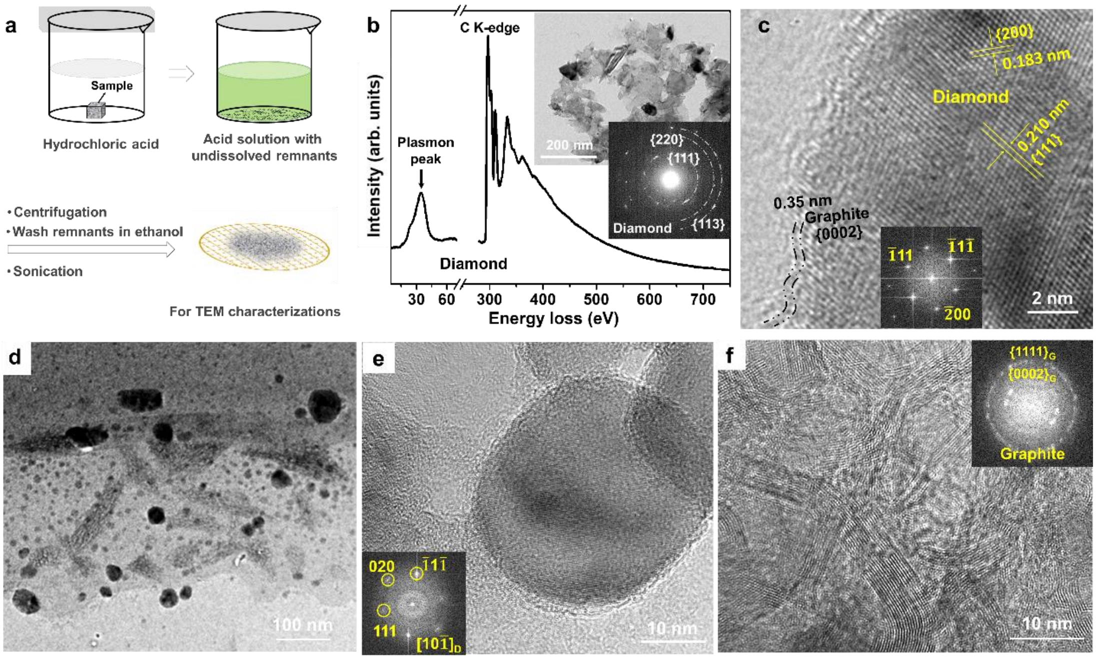

Supplementary Figure 5. Extraction of DNPs from furnace samples. a Schematic of the extraction process. **b** EELS spectrum of extracted DNPs. Insets show the TEM image and corresponding electron diffraction patterns of the re-aggregated DNPs after being extracted from the carburizing steel sample. **c** High-resolution TEM image and the corresponding Inverse Fast Fourier Transformation (IFFT, inset) of an extracted

DNP with a thin graphite layer. **d** Large numbers of DNPs on the support carbon film of the TEM grid. **e**, High-resolution TEM images and the corresponding IFFT (inset) of an individual DNP. **f** High-resolution TEM image of the tangled graphite nanoribbons from undissolved remnants and the corresponding IFFT (inset). See Methods section 'Extraction of DNPs inside bulk samples' for more details.

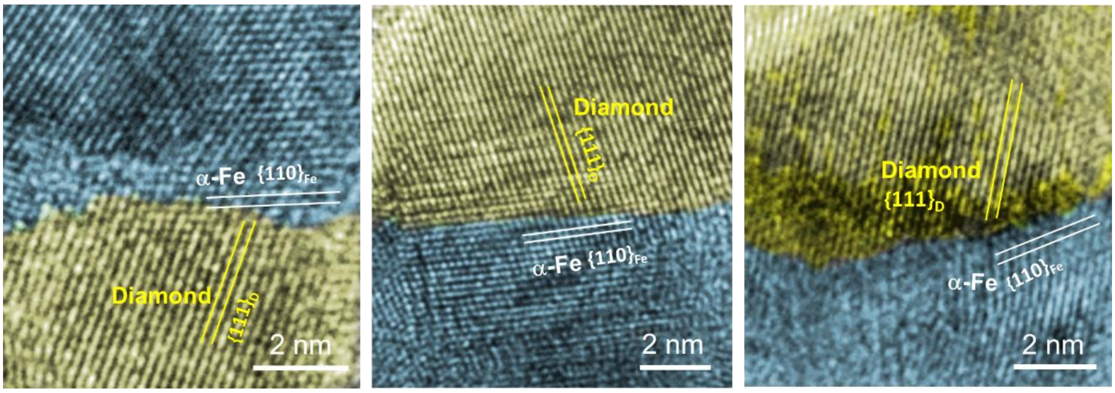

Supplementary Figure 6. High-resolution TEM images of the typical interfaces between diamond (yellow) and the iron matrix (blue) in the Fe-DNPs sample.

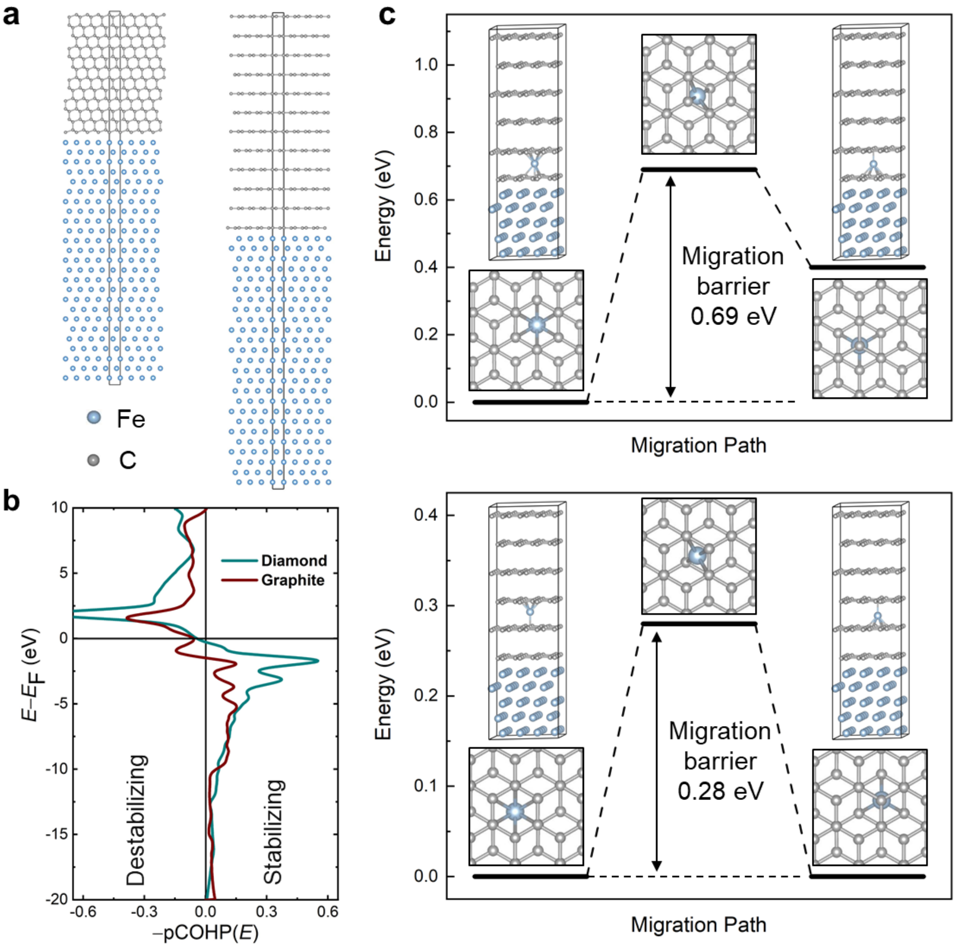

Supplementary Figure 7. DFT modeling of Fe-diamond/graphite interfaces and NEB predictions for Fe migration. a The relaxed Fe-diamond ($\{110\}_{Fe}$-$\{111\}_{D}$) and Fe-graphite ($\{110\}_{Fe}$-$\{0002\}_{G}$) interface models. The dashed box marks the two unit cells (both contain 25 Fe and 24 C atoms). The spacings of the relaxed structures, ~0.19 nm for $\{110\}_{Fe}$ and ~0.20 nm for $\{111\}_{D}$, match well with the experimental ones in Fig. 3e. The atomic models are shown in Fig.4b. **b** The projected COHP (-pCOHP) onto the two Fe-C interfaces. Stabilizing bonding interactions are found at almost all the occupied bands for the Fe-C bond pairs, indicating strong coupling of the Fe-diamond interface. Some destabilizing antibonding contributions are found at and right below the Fermi level for the Fe-C bond pairs at the graphite interface, indicating less strong Fe-C chemical bonds. The Fe-graphite interface has a much shorter interatomic distance ~0.21 nm than the ~0.36 nm between (0002) graphite planes, and the latter are expected to serve as the easy channels for the migrating Fe atoms. **c** The Fe migration path and energy barrier predicted by NEB calculations, for the diffusional hopping of Fe atom along the channels in-between graphite (0002) planes. The top and bottom panels represent the migration in the first and second interlayer gap next to the iron matrix, respectively. Note that the two graphite layers making up the first gap are no longer identical due to the strong bonding of one graphite layer with the bulk Fe matrix, leading to an energy rise to 0.69 eV in the migration barrier from the 0.28 eV in the second graphite gap.

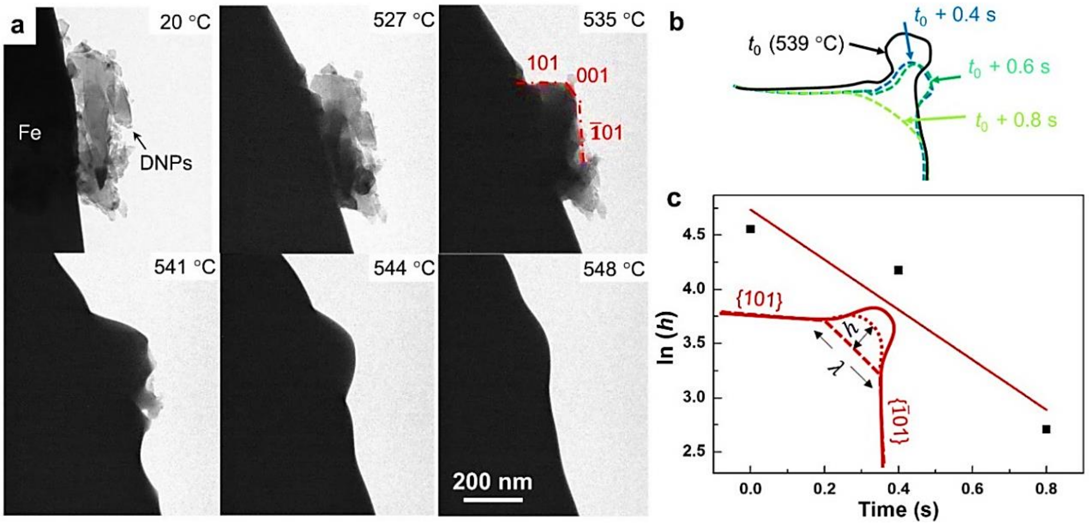

Supplementary Figure 8. Calculation of the surface diffusivity based on the morphological change in the in-situ TEM observation. a Real-time recording of the entrance process medicated by surface diffusion of iron atoms over a DNP aggregate (see Supplementary Movie 4). **b** Two-dimensional outlines change of the surrounded DNP aggregate by iron flux with time. **c** The measured retreating distance *h* of the {110} faceted surface versus time. λ is the corresponding segment length of the formed single-hump (as illustrated in the inset).

Fe-C Gibbs free energy diagram

The Gibbs free energy curve of Fe-C at 1,253 K (Supplementary Fig. 9) is calculated using the Thermal-Calc software. The thermodynamic data of the Fe–C system was critically assessed by Gustafson using the CALPHAD (Calculation of Phase Diagrams) method[3].

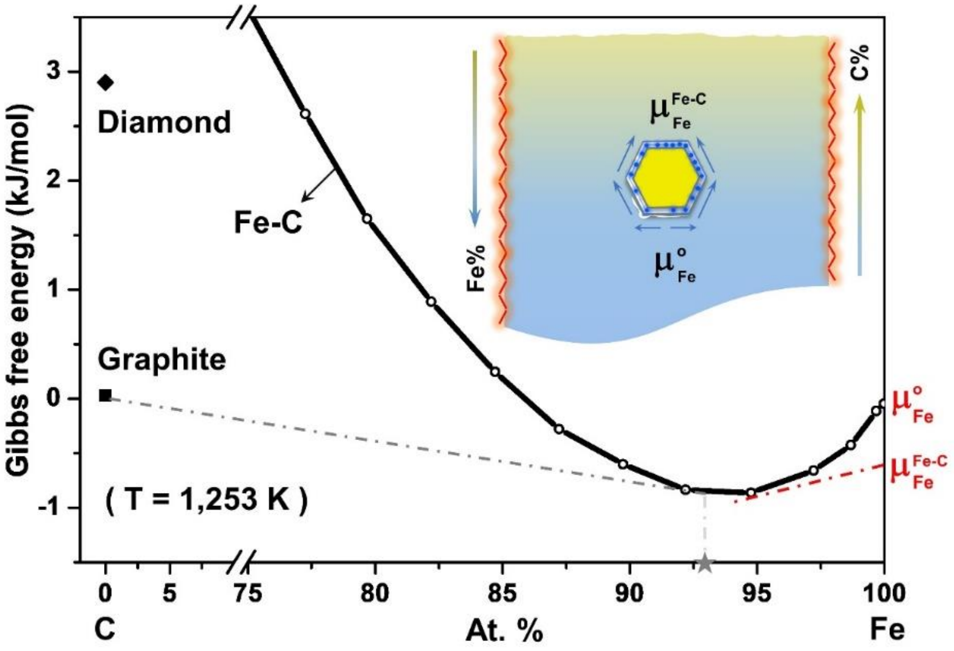

Supplementary Figure 9. The calculated Gibbs free energy curve of Fe-C at 1,253 K. The equilibrium solubility (~7 at. %) of carbon in γ-Fe at 1,253 K is given by the carbon concentration at the common tangent (dash dotted lines) touch point on the Gibbs free energy curve (solid curve). At any carbon concentration, one can find the chemical potential (μ_{Fe}) of Fe in that Fe-C solid solution from the intercept of the tangent line of the Gibbs free energy curve with the *y* (right) axis; an example, given in the figure, indicates that the chemical potential of Fe in the solution would be lower relative to that of pure Fe. The schematic inset demonstrates a DNP (in yellow) inside iron (in blue). Fe atoms flow from the location with higher chemical potential (underneath the particle) to that with lower chemical potential (above the particle) via the graphite channel on the DNP surface in contact with iron.

Supplementary Discussion

I. Initial sink in process: Fe surface diffusion to wrap around DNP

An effective surface diffusion coefficient D_S of $\sim10^{-10}$ cm^2/s can be estimated from our real-time observations of the morphology changes (see Supplementary Fig. 8a). By drawing an analogy to the flattening of an arched surface (Supplementary Fig. 8b), a

quantitative two-dimensional surface diffusion model to estimate D_S was formulated following the method in refs[4,5]. Assuming that the iron over DNPs is single-hump, the reference can be chosen as the final rest position of the {110} facets of iron, which have the minimum surface energy. The height (h) change of the receding facet is then measured as a function of time. As plotted in Supplementary Fig. 8c, the natural logarithm of h is an approximately linear function of time, giving a slope of S. The surface diffusivity D_S can then be estimated using the following equation[5]:

$$D_s = -\frac{Sk_B T}{4\gamma_M \Omega^2 \kappa}\left(\frac{\lambda}{2\pi}\right)^4 \quad (1)$$

where k_B is the Boltzmann constant, temperature T is 810 K, γ_M is the surface energy of iron ($\gamma_{Fe(110)} = 2$ J/m^2), Ω (atomic volume of Fe) is 0.0117 nm^3, κ is the surface atomic density (=17.2 atoms/nm^2) , or $\Omega^2\kappa = L^4$, where L is interatomic distance, and λ is the segment length of the hump in the rest position (the measured value of 90 nm). This analysis yields a calculated $D_S = 5.7\times10^{-10}$cm^2/s. This magnitude is consistent with the reported surface diffusivity of α-iron at 810 K (3.8×10^{-10} cm^2/s) [6], confirming that it is surface diffusion that wraps Fe around the DNPs.

II. MC-simulated motion of DNP inside Fe

We use Monte Carlo (MC) simulation to demonstrate that at temperature T~1,200 K the transport of Fe around the DNP is able to sustain the DNP motion. Considering that $v \propto \exp(\Delta E_{\text{Fe-in-graphite}}/k_B T)$, $v \propto \nabla\mu$ and $v \propto d^{-1}$, our extrapolation suggests a v of ~25 nm/s for a DNP d = 100 nm in diameter under the maximum chemical potential difference (~0.005 eV/atom across its two poles, see Supplementary Fig. 8). This estimated DNP velocity is close to what was observed in experiments (the DNPs reach the maximum depth of ~1 mm within 5 h, the average motion velocity is hence ~0.2 mm/h or ~50 nm/s).

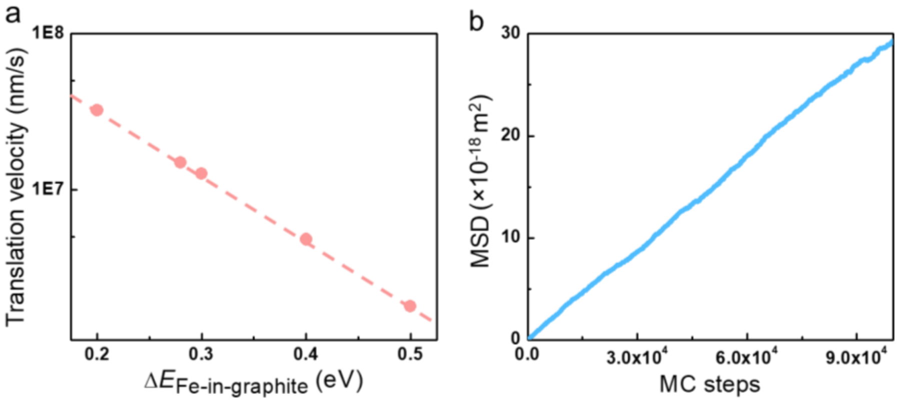

Supplementary Figure 10. Monte Carlo simulation of the DNP motion and Fe atomic diffusivity. a The motion velocity (v) of a DNP (d = 5 nm) at 1,200 K for different $\Delta E_{\text{Fe-in-graphite}}$ (the migration barrier of an isolated Fe atom diffusing in between graphite layers) under $\nabla\mu$ = 0.01 eV per interatomic distance. The dashed line indicates the fitting of $v \propto \exp(\Delta E_{\text{Fe-in-graphite}}/(k_B{*}1200))$. Based on these data, extrapolations can be made for other $\nabla\mu$ and d values. **b** Mean squared displacement (MSD) versus MC steps during the MC simulation of an isolated Fe atom diffusing in the first van der Waals gap using $\Delta E_{\text{Fe-in-graphite}}$ = 0.69 eV at 1,200 K. The effective diffusivity $D_s^{'}$ used in in the following Equation (4) is derived from the slope of MSD.

III. An analytical estimate of the velocity of DNP inside Fe and the local stress on DNP

In addition to the extrapolation from the direct MC simulations of DNP motion discussed above, we have also performed a back-of-the-envelope numerical estimate of the travel velocity of the DNP based on the mass conservation. Basically, all the Fe atoms transport from underneath the DNP need to get incorporated into the expanding Fe lattice above the DNP. These Fe atoms come as interstitials, but accumulate to form new lattice sites.

If the DNP is treated as a sphere with diameter d, and the transported Fe mass flux $M_{\text{Fe-up}}$ (in the unit of g/s) through the equatorial plane of the diamond sphere can be written as

$$M_{\text{Fe}-up} = \cdot D_s^{'} \cdot (\Delta c / d) \cdot (\pi d \cdot \delta_s) \cdot \Omega \cdot \rho \qquad (2)$$

where $D_s^{'}$ is the effective diffusivity of Fe atoms at the Fe-DNP interface, Δc is the concentration difference of Fe across the DNP, δ_s is the thickness of Fe atoms flux and equals the diameter of a Fe atom, about 0.25 nm, and Ω (atomic volume of Fe) is 0.0117

nm^3. The downward movement of the DNP fills in the space left by the transported Fe atoms, and the mass flux of Fe atoms M_{Fe} can also be written as

$$M_{\mathrm{Fe}} = v \cdot (\pi d^2 / 4) \cdot \rho$$

(3)

By $M_{\mathrm{Fe\text{-}up}}$=$M_{\mathrm{Fe}}$, the translation velocity v can be expressed as:

$$v = 4\delta_{\mathrm{s}} \cdot \Delta c \cdot D_{\mathrm{s}}^{'} \cdot \frac{\Omega}{d^2} \quad (4)$$

The value of $D_{\mathrm{s}}^{'}$ is not available in experiments. So, we performed a two-dimensional MC simulation (analogous to isolated Fe diffused within a graphite van der Waals gap) using the migration energy barrier of 0.69 eV (Fig. 4c). The corresponding mean squared displacement (MSD) versus MC steps at 1,200 K is shown in Supplementary Fig. 10b, leading to $D_{\mathrm{s}}^{'}$ ~6×10^{-11} m^2/s; for the smaller migration energy barrier of 0.28 eV (Fig. 4c), the $D_{\mathrm{s}}^{'}$ can reach ~3×10^{-9} m^2/s. For a DNP with d = 100 nm, when the Fe concentration difference across its two poles is as small as 0.01 at.%, the estimated velocity of DNP can reach to ~10 nm/s, which is in the same order as what was observed in experiments, ~50 nm/s.

After the DNP has fully immersed into the iron crystal, a local stress arising from the chemical concentration difference replaces the capillary force to push it down further, i.e., the chemical potential driving force is spent in overcoming the lattice friction during the displacive motion of DNPs via the local stress. The local stress normal to the Fe-DNP interfacial boundary is generated when upward Fe atoms are trying to squeeze into the upper interface. We performed a back-of-the-envelope numerical estimate of the maximum value of the local stress by equating the chemical-concentration-gradient-driven atomic Fe flux ($J_{\mathrm{c}} = D_{\mathrm{s}}^{'} \cdot \frac{dc}{dz}$) to the local stress difference induced the atomic flux (J_σ).

The atomic flux J across the diamond nanoparticle surface layer can be written as[7]

$$J = \frac{N}{\Omega} \frac{D_{\mathrm{s}}^{'}}{k_B T} (\nabla \mu) \quad (5)$$

where k_B is the Boltzmann constant; T is the temperature; N is the number of diffusing atoms; $\nabla\mu$ is the chemical potential gradient. For the stress difference induced chemical potential gradient, $\nabla\mu$ can be expressed as[8]

$$\nabla\mu = \Omega\frac{d\sigma}{dx} \tag{6}$$

Therefore, the local stress difference ($\Delta\sigma$) induced the atomic flux across the diamond nanoparticle with the diameter of d (J_σ, in the unit of atoms #/m^2/s) can be calculated as

$$J_\sigma = \frac{D_s^{'}}{k_B T}\left(\frac{\Delta\sigma}{d}\right) \tag{7}$$

where $\Delta\sigma$ stands for the stress difference across the diamond sphere to sustain the atomic flux, d is the diameter of DNP. If $J_c = J_\sigma$, the $\Delta\sigma$ is estimated as

$$\Delta\sigma = \frac{dc}{dz}\cdot k_B \mathrm{T}\cdot d \tag{8}$$

For a DNP with d = 100 nm, with the maximum chemical concentration difference across its two poles (at 1,250 K, the maximum carbon concentration reaches 1.8 wt. %), Equation (8) predicts a maximum local stress σ~120 MPa.

IV. Graphite layers on DNPs surfaces

Graphite is more stable than diamond with a Gibbs energy reduction of ~30 meV/atom[9]. As Fe is a highly effective catalyst to facilitate the transformation from diamond to graphite[10-12], it is not surprising to observe a thin graphite layer at the carburizing temperatures when the DNPs get wet by the arriving Fe flux. As shown using the example in Fig.2d, the surface of the DNP is enclosed by a layer of graphite before embarking on the journey into iron. The core-loss EELS spectra displayed in Fig.2c shows a small carbon pre-peak (π*) at ~285 eV typical for graphitic material[13], as a result of the graphite sheath on DNPs.

The average size of DNPs becomes smaller as they move progressively into iron (Supplementary Fig. 4). Apparently, some carbon gradually leaves the surface as the

DNP intrudes deeper and deeper into Fe. This happens because the graphite layers on the DNP surface can scratch off due to their weak van der Waals interplanar bonding[14], and the carbon atoms would dissolve into iron at the carburizing temperatures to satisfy the solubility[11,12] (decreasing the rate of graphitization of diamond[15]). This explains the gradual decrease in the size of DNPs (Supplementary Fig. 4). For some DNPs that have traveled deep into iron, no graphite layers remain detectable on their circumference (Supplementary Fig. 5e), and the diamond/Fe interface then becomes atomically sharp and semi-coherent (Fig.3e). When that happens, we expect the DNPs to hit a limit, in terms of their minimum size and the maximum travel distance. The reason for this maximum depth is that the graphitization on the DNP surfaces would no longer continue. The Gibbs energy difference between diamond and graphite at 1,253 K is a function of pressure[16], see Supplementary Fig. 11a. For the small DNP embedded inside iron, any graphitization of its surface would incur a rather large expansion in volume, which leads to high pressure (Supplementary Fig. 11b), and hence stiff energy penalty disfavoring the transformation into graphite. The absence of the graphite at the strongly bonded Fe/DNP interface takes away the diffusion channels for the Fe flux. The DNPs would then stay put and their forward-motion practically halts.

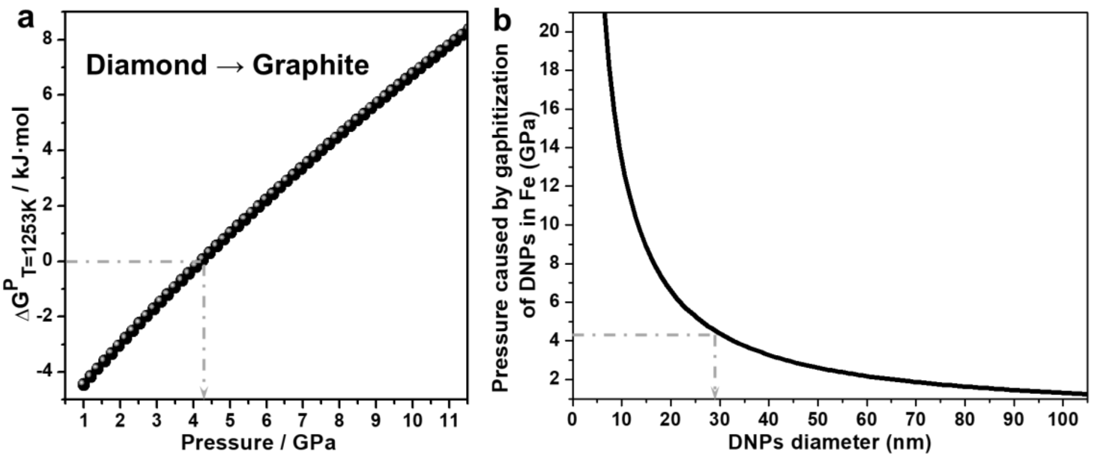

Supplementary Figure 11. Gibbs free energy change and pressure caused by graphitization of DNPs inside iron. a Gibbs free energy change for the transformation from diamond to graphite (at the constant temperature of 1,253 K) as a function of pressure. The critical transition pressure is about 4.3 GPa, above which it is no longer energetically favorable to transform the diamond into graphite. Take a DNP with the diameter of 100 nm inside iron for example (note that the DNP is assumed to be a sphere

here). If 10 atomic layers of its surface have turned into graphite, the volume expansion ratio is about 8.6 %, which, multiplying the bulk modulus (155 GPa) of FCC iron at 1,253 K, gives a pressure of about 13 GPa. This is much higher than the critical transition pressure for the diamond→graphite transformation to occur. If two atomic graphite layers are generated, the pressure is about 4 GPa, and the graphitization remains energy favorable. **b** The pressure caused by graphitization of DNPs inside iron, as a function of the DNP diameter. The smaller DNPs inside iron, the higher pressure caused by their surface graphitization, and the more difficult for them to be graphitized further. Note that DNPs used in our experiments are actually not perfect spheres, so the predicated critical transition size here has a certain discrepancy with the actual value.

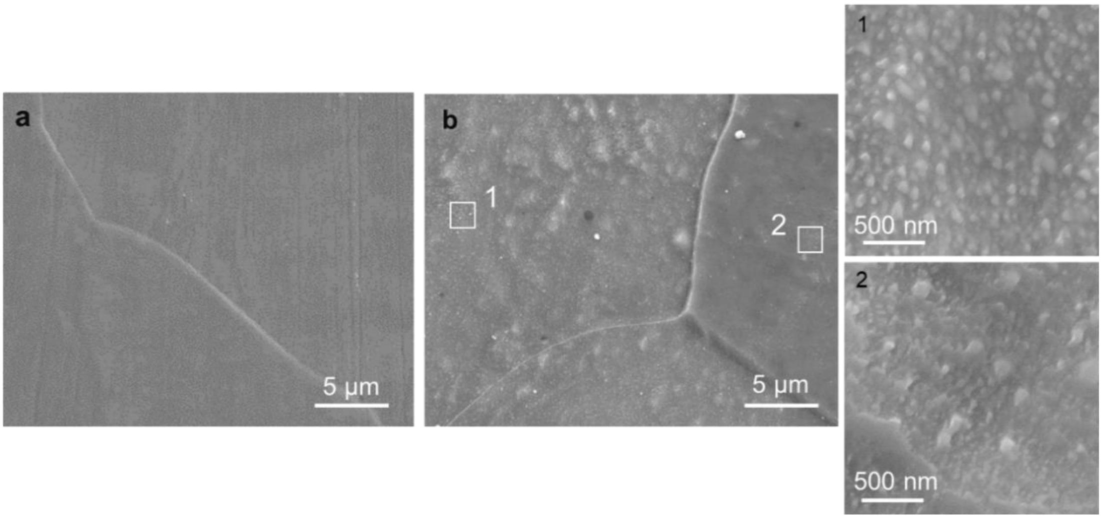

Supplementary Figure 12. Comparison of the deeply etched surface with and without DNPs. a SEM image of the deeply-etched original sample with a relatively smooth surface. **b** SEM image of the deeply-etched DNPs-iron sample and the SEM view of two enlarged zones from different grains with different etching depths. Note that these DNPs are not concentrated at grain boundaries of iron and no obvious DNPs-free regions near the grain boundary have been found.

V. Nanoindentation and nanoscratching tests

The nano-hardness as well as coefficient of friction (COF) of pure iron and Fe-DNPs samples (cooled in furnace or water) were measured using the Brooker-Hysitron TI 950 TriboIndenter, whereby the indentation was performed with forces of 1 0,000 μN by a Bercovich indenter and the nanoscratching was carried out by a conical diamond tip (10 μm in diameter) under a constant normal loading of 20 mN. Before nanoscratching test, a prescan with a low normal force of 0.2 mN was performed to get the initial surface information (height and roughness) of the tested specimens, which will be used to correct the final scratch depth (named tilt correction). After the tilt correction, the

sample was scratched for 50 μm (2 μm/s) to obtain the scratch depth and coefficient of friction along the tested lateral distance. As shown in Supplementary Fig. 13, the hardness, elastic modulus and COF of the Fe-DNPs sample have changed markedly in comparison with the iron matrix, especially for the Fe-DNPs sample quenched in water, of which the nanohardness is as high as ~15 GPa and the COF value is halved. Note that the hardness improvement of Fe-DNPs sample is not dominant by the carbon content increase but the introduction of DNPs, since the reported nanohardness of the Fe-C pearlite and martensite phases are, respectively, ~2.5 GPa[17] and 5~10 GPa for martensite with different carbon contents[18,19], both lower than the hardness measured in the Fe-DNPs samples.

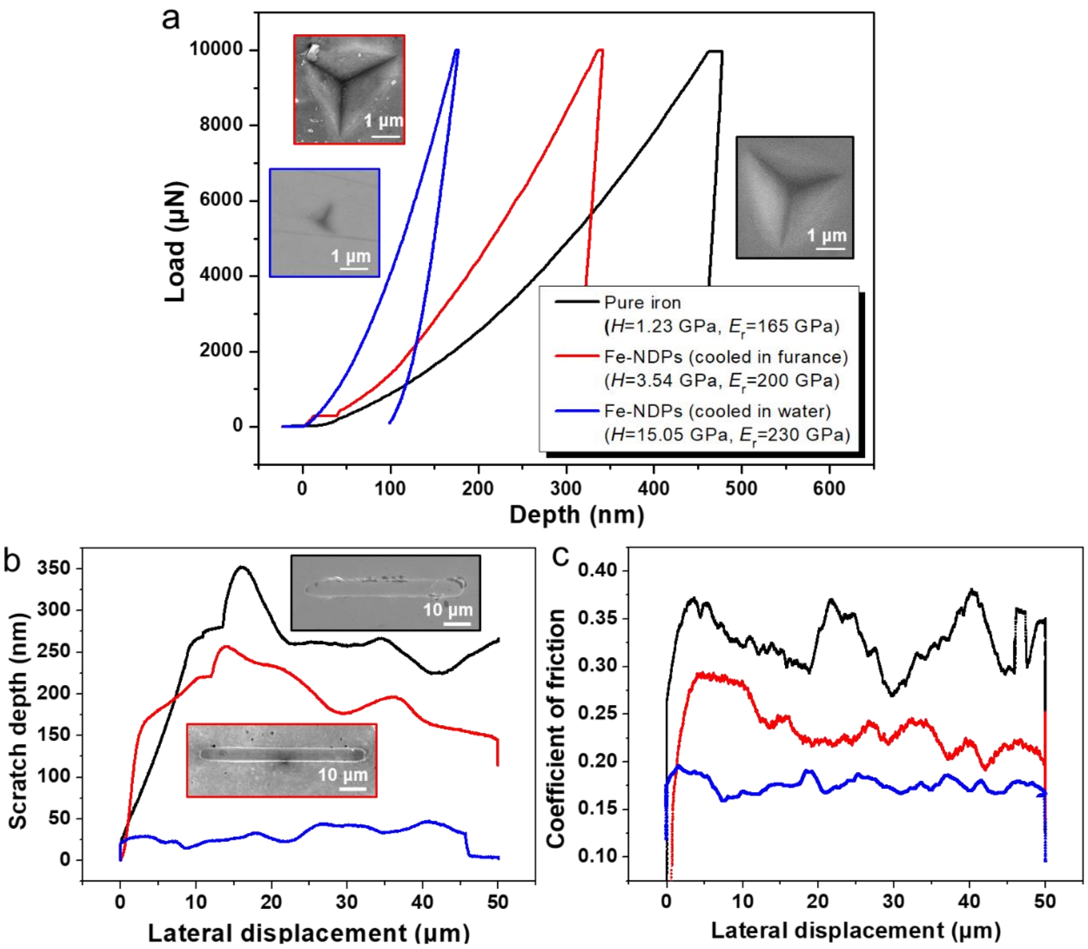

Supplementary Figure 13. Remarkably improved hardness and decreased coefficient of friction of the quenched Fe-DNPs sample. a The typical load-displacement curves of pure iron (black), Fe-DNPs samples cooled in furnace (red) and in room temperature water (blue). Insets display the SEM images of indentations on the three samples. The scratch depth **b** and COF **c** of the tested specimens as a function of

the lateral displacement of the diamond tip. Inserted SEM images in **b** show the scratch morphology of pure iron and Fe-NDPs (cooled in furnace) samples. The scratch depth of the Fe-DNPs sample cooled in water is so small that it is not observable in the micrograph.

Supplementary References

1. Wang, X., Zhang, S., Li, J., Xu, J. & Wang, X. Fabrication of Fe/Fe3C@porous carbon sheets from biomass and their application for simultaneous reduction and adsorption of uranium(VI) from solution. *Inorganic Chemistry Frontiers* **1**, 641-648 (2014).
2. Doeff, M. M. *et al.* Impact of carbon structure and morphology on the electrochemical performance of LiFePO4/C composites. *Journal of Solid State Electrochemistry* **12**, 995-1001 (2008).
3. Gustafson, P. A thermodynamic evaluation of the Fe–C system. *J. Metall.* **14**, 259–267 (1985).
4. Xie, D. *et al.* In situ study of the initiation of hydrogen bubbles at the aluminium metal/oxide interface. *Nature Materials* **14**, 899-903 (2015).
5. Mullins, W. W. Flattening of a nearly plane solid surface due to capillarity. *J. Appl. Phys.* **30**, 7 (1959).
6. Blakely, J. M., & Mykura, H. Studies of vacuum annealed iron surfaces. *Acta Metallurgica* **11**, 5 (1963).
7. Tian, L., Li, J., Sun, J., Ma, E., & Shan, Z. W. Visualizing size-dependent deformation mechanism transition in Sn. *Scientific Reports* **3**, 2113 (2013).
8. Tu, K. N. Recent advances on electromigration in very-large-scale-integration of interconnects. *J. Appl. Phys.* **94**, 5451-5473 (2003).
9. Shin, H., Kang, S., Koo, J., Lee, H. & Kwon, Y. Cohesion Energetics of Carbon Allotropes : Quantum Monte Carlo Study. *Journal of Chemical Physics* **140**, 114702 (2014).
10. Peçanha, M. P., & Filgueira, M. The catalytic effect of iron on the graphitization of diamonds. *Int. J. Mater. Res.* **104**, 794-798 (2013).
11. Narulkar, R., Bukkapatnam, S., Raff, L. M., & Komanduri, R. Molecular dynamics simulations of diffusion of carbon into iron. *Philos. Mag.* **88**, 1259-1275 (2008).
12. Zenkin, S., Gaydaychuk, A., Okhotnikov, V., & Linnik, S. CVD Diamond Interaction with Fe at Elevated Temperatures. *Materials* **11**, 2505 (2018).
13. A. Ferrari, J. R. Interpretation of Raman spectra of disordered and amorphous carbon. *Phys. Rev. B.* **61**, 14095-14107 (2000).
14. Charlier, J., Gonze, X. & Michenaud, J. Graphite interplanar bonding : electronic delocalization and van der Waals interaction. *Europhys. Lett.* **28**, 403-408 (1994).
15. Naidich, Y. I., Umanskii, V. P., & Lavrinenko, I. A. *Strength of the diamond-metal interface and brazing of diamonds*. Vol. 3 104-123 (Cambridge Int Science Publishing, 2007).
16. Li, W. S., Zhang, J., Dong, H. F., Chu, K., Wang, S. C., Liu,Y. & Li, Y. M. Thermodynamic and kinetic study on interfacial reaction and diamond graphitization of Cu Fe-based diamond composite. *Chinese Physics B* **22**, 018102 (2013).
17. Kochmann, W., Reibold, M., Goldberg, R., Hauffe, W., Levin, A. A., Meyer, D. C., ... & Paufler, P. (2004). ., 372(1-2), L15-L19. Nanowires in ancient Damascus steel. *Journal of Alloys and Compounds* **372**, L15-L19.
18. Ohmura, T., Tsuzaki, K., & Matsuoka, S. Nanohardness measurement of high-purity Fe–C martensite. *Scripta Materialia* **45**, 889-894 (2001).
19. Zhang, L., Ohmura, T., & Tsuzaki, K. . Application of nanoindentation technique in martensitic

structures. *Nanoindentation in Materials Science*, 109-130 (2012).
