# Supplementary material for: Inward motion of diamond nanoparticles inside an iron crystal
Source: Nat Commun. 2024 May 31;15:4659. doi: 10.1038/s41467-024-48692-5 (PMC11143255; doi:10.1038/s41467-024-48692-5)
Supplement: Supplementary file 3 — Description of Additional Supplementary Information [file 41467_2024_48692_MOESM3_ESM.pdf]

Description of Additional Supplementary Information

Supplementary Movie 1 An in-situ TEM video showing sinking-in process of an individual diamond aggregate into a high-purity iron lamella (480 oC~550 oC). The video was recorded at 10 frames/second, and played at the original speed. Snapshots of this video are shown in Fig. 2 in the main text.

Supplementary Movie 2 Initial sinking-in process of DNPs sitting on the surface of a low-carbon steel lamella inside the STEM. The bright-field STEM imaging (left panel) and the simultaneous secondary electron (SE) imaging (right panel) the same zone. The movie is speeded up ×5.

Supplementary Movie 3 Real-time recording of the engulfed process of diamond nanoparticles by the iron flux. The movie was recorded at 5 frames/second, and played at 10× speed. Snapshots of this movie are shown in Supplementary Fig. 8.

Supplementary Movie 4 Monte Carlo simulated motion process of a diamond nanoparticle inside iron under the chemical potential gradient $\nabla\mu = 0.01$. Snapshots of this movie are shown in Fig. 4d.
